# Vitamin D levels among HIV-infected patients – peculiar features

**DOI:** 10.1186/1471-2334-13-S1-P11

**Published:** 2013-12-16

**Authors:** Carmen Chiriac, Iringo Erzsébet Zaharia-Kezdi, Brînduşa Ţilea, Anca Georgescu, Cristina Gîrbovan, Andrea Incze, Nina Șincu, Andrea Fodor

**Affiliations:** 1 Clinic of Infectious Diseases, University of Medicine and Pharmacy Tîrgu Mureş, Romania; 2 Emergency Clinical County Hospital Tîrgu Mureş, Romania

## Background

25-hydroxyvitamin D (25[OH]D) plays an essential role in the homeostasis of calcium and bone metabolism, but it is acknowledged as an immunomodulator as well. Several studies have described a high prevalence of 25[OH]D deficiency in HIV-infected patients, as well as its role in predicting disease progression.

## Methods

We performed a descriptive, cross-sectional study on a group of 68 HIV-infected patients monitored in the Regional HIV/AIDS Centre Mureş, during December 2012-February 2013. We determined plasma levels of 25[OH]D. We estimated the proportion of patients with 25[OH]D deficiency/insufficiency and correlated 25[OH]D levels to HIV-RNA plasma viral load, CD4+ T-cells count and associated pathology. Data were compared to those obtained from a group of healthy, HIV-negative volunteers. Statistical analysis was performed using the GraphPad program.

## Results

We studied 68 C-stage HIV-infected patients, 40 male: 28 female, with an average age of 26 and a median of 23. The average plasma 25[OH]D level was 30.94 ng/mL, median 30 ng/mL in HIV-positive patients compared to an average of 44.72 ng/mL and median 39.82 ng/mL in HIV-negative subjects (p=0.0109). 25[OH]D deficiency <20 ng/mL was found in 23.52% HIV-infected patients, while 26.47% HIV-positive subjects had medium levels of 25[OH]D insufficiency, between 20-30 ng/mL. Only 2 patients had severe 25[OH]D deficiency, below 10 ng/mL. We did not find any statistically significant correlations of 25[OH]D levels with CD4+ T-cells level, HIV-RNA plasma viral load or protease inhibitor therapy. Patients with HIV-tuberculosis co-infection had significantly lower levels of 25[OH]D (p=0.0454) than HIV-positive subjects without tuberculosis.

## Conclusion

25[OH]D plasma levels are lower in HIV-infected patients compared to the general population. Low 25[OH]D levels are reported in patients with HIV-tuberculosis co-infection. Low 25[OH]D levels may indicate unfavorable outcome in HIV-infected patients.

